# An Unusual Case of Lynch Syndrome

**DOI:** 10.7759/cureus.62420

**Published:** 2024-06-15

**Authors:** Rita Pinheiro Duque, Nuno Santos, Bárbara Freire, Carlos M Oliveira, João M Mendes, Juliana P Macedo, Francisco Sampaio

**Affiliations:** 1 General Surgery, Unidade Local de Saúde do Médio Ave, Vila Nova de Famalicão, PRT

**Keywords:** colorectal cancer, immunocompetent patient with necrotizing fasciitis, hereditary diseases, lynch syndrom, celulitis

## Abstract

Lynch syndrome is the most common cause of hereditary colorectal cancer. It usually develops asymptomatically until symptoms related to colorectal carcinoma appear, such as gastrointestinal bleeding, abdominal pain, and changes in bowel habits and/or stool characteristics. Oftentime, when these clinical signs and symptoms are not present, the diagnosis becomes challenging. We present the clinical case of a 69-year-old woman, adopted, with no known previous history, who presented to the emergency department with low back pain, without irradiation, that had been going on for three days, associated with inflammatory signs in the right hip region. There were no urinary or sensory alterations and no recent trauma. She was initially discharged with antibiotherapy with the diagnosis of hip cellulitis. As the symptoms continued and the inflammation spread to the right lower limb, she returned to the emergency department. A CT scan revealed an abscess (17 cm) in the right buttock, complicated by necrotizing fasciitis due to fistulization from a tumor in the right colon. She underwent an exploratory laparotomy, which identified a neoplasm of the ascending colon, adherent to the abdominal wall, in the right lumbar region. Right hemicolectomy and drainage of the right buttock/thigh abscess were performed. The histology was compatible with invasive adenocarcinoma, with high-grade dysplasia but well differentiated, pT3G1N0. The immunohistochemistry was suggestive of Lynch syndrome.

## Introduction

Lynch syndrome is the most common cause of hereditary colorectal cancer (accounting for 1-3% of all colon neoplasms) and is associated with a mutation in DNA mismatch repair genes and microsatellite instability [1]. It has an autosomal dominant transmission and is characterized by the appearance of colorectal carcinoma at a young age (40-48 years) [2,3]. The presentation of this pathology can be divided into colonic and extra-colic manifestations [4]. Most patients are asymptomatic until symptoms related to colorectal carcinoma appear, such as gastrointestinal bleeding, abdominal pain, and changes in bowel habits [4,5]. There is a high probability of synchronous and metachronous tumors in this syndrome, and around 7% of patients may have more than one cancer at the time of diagnosis [6,7]. Among the various extra-colic manifestations, the most common is endometrial cancer [4,5]. There have also been reports of neoplasms of the ovary, stomach, small intestine, pancreas and bile ducts, genitourinary system (kidney, ureter, and bladder), brain (glioma), and various skin diseases [8,9]. Family suspicion of Lynch syndrome is based on the Amsterdam II criteria, which state that there must be a history of colorectal carcinoma or other cancers that can be associated in three or more members, affecting two generations, with one of the neoplasms diagnosed before the age of 50 [3]. Below, we present a clinical case of an atypical clinical presentation of Lynch syndrome and a review of the literature on the subject.

## Case presentation

A 69-year-old woman, with no previous history of serious health problems, came to the emergency department with low back pain, without irradiation, which had been going on for three days. There was no history of trauma and no urinary or sensory alterations. On physical examination, she was apyretic, with no hemodynamic alterations. There was pain on palpation of the lumbar apophyses and right paravertebral region, with hardening of the skin and redness in the buttock region. After analgesia with diclofenac and thiocolchicoside, she underwent a lumbar X-ray, which revealed no alterations, and clinical analyses that showed leucocytosis (31.56 x 109/L) and an increase in C-reactive protein (34.7 mg/L). After an evalution in the General Surgery office, a diagnosis of cellulitis of the buttock was made. She was discharged home with antibiotic therapy (ciprofloxacin).

**Figure 1:**
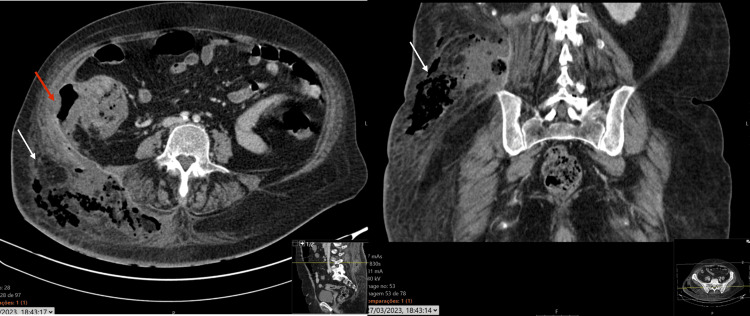
CT scan of the abdomen and pelvis The white arrows show the area of necrotizing fasciitis, with gas bubbles in the subcutaneous cellular tissue. The red arrow shows the area of fistulization of the right colonic lesion.

Two days later, due to continued and worsening symptoms, the patient returned to the emergency department. She presented with cellulitis of the right buttock with extension to the lumbar region, with flictenas, under tension. The condition was compatible with necrotizing fasciitis. In this context, she underwent a CT scan (Figure 1), which revealed an oval abscess of 17 cm in the right buttock region, with an extensive emphysematous component and a small amount of fluid, related to an infectious process of the phlegmonous/gangrenous type. This lesion appeared to result from the fistulization of a perforated tumor in the right colon into the soft tissues of the buttock and right abdominal wall. She underwent an emergency exploratory laparotomy, where the abscess on the right buttock was drained and necrotizing fasciitis found in the area was debrided. A neoplasm of the ascending colon was visualized, adherent to the abdominal wall, at the level of the right lumbar region. There was no encroachment on any other neighboring structures, no peritoneal carcinomatosis, and no palpable liver metastases. A right hemicolectomy was performed with latero-lateral ileo-colic anastomosis. The surgical specimen was sent for pathological examination. During hospitalization, she underwent a course of antibiotic therapy with piperacillin-tazobactam. Recovery was complicated by evisceration of the surgical wound, which was corrected surgically and with negative pressure therapy.

Histology of the surgical specimen confirms a diagnosis of invasive adenocarcinoma of the cecum/ascending colon with high-grade dysplasia but well differentiated, margins without tumor, pTMN T3G1N0. Immunohistochemical evaluation revealed that it was Lynch syndrome. The follow-up was made in a general surgery appointment. A staging CT scan and colonoscopy showed no distant disease. Mammography and pelvic MRI ruled out gynecological pathology. In an oncology group consultation, it was decided to carry out adjuvant chemotherapy.

## Discussion

Lynch syndrome is an important cause of colorectal cancer and is hereditary. The Amsterdam II criteria make it possible to assess the familial suspicion of this syndrome. These criteria could not be applied to the clinical case presented above due to the lack of information about the patient's relatives, as she was adopted and had not met her biological family.

Unlike sporadic colorectal carcinoma, Lynch syndrome carcinoma is more likely to be located in the right colon [7], which is the case here. It mostly originates from adenomatous polyps, which have a higher degree of dysplasia and/or villous histology than sporadic polyps [7].

In the clinical case presented, the initial manifestation of colorectal carcinoma differs from those described in the literature. It was a necrotizing fasciitis of the right thigh, following fistulization of the primary tumor. The patient denied any kind of visible hemorrhage, as well as alterations to her intestinal transit, which made it difficult to diagnose the cancer itself.

The extra-colic manifestations of Lynch syndrome justify an imaging examination for staging, in this case, a thoraco-abdomino-pelvic CT scan (which revealed no alterations), as well as a gynecological assessment. In addition, there is a greater risk of breast pathology [9], which was not found in the mammogram carried out. It should be noted that the late diagnosis of this colorectal carcinoma may be due to a lack of information about the patient's biological family, since the suspicion of a hereditary disease was never raised and the necessary genetic assessment was never carried out. The fact that the patient had never been screened for colorectal cancer as recommended by the NHS also contributed to the progression of the neoplasm and prevented its timely diagnosis. Screening for Lynch syndrome now begins in the offspring of the index case, who are targeted for genetic evaluation. Ideally, in the event of a positive mutation, screening with colonoscopy should be started up to 10 years before the age of the youngest known case or between 20 and 30 years [10]. If there is no mutation, family members should follow the normal screening age for sporadic colorectal carcinoma.

## Conclusions

This clinical case reminds us that sometimes the most obvious diagnosis may not be the right one. In fact, we should be aware of other, less common forms of presentation of diseases we thought we knew about. Lynch syndrome remains the main cause of hereditary colonic neoplasia. This condition makes screening in families with a known mutation imperative, making it possible to prevent, recognize, and treat these neoplasms in good time.
